# The Insulin/IGF Signaling Regulators Cytohesin/GRP-1 and PIP5K/PPK-1 Modulate Susceptibility to Excitotoxicity in *C. elegans*

**DOI:** 10.1371/journal.pone.0113060

**Published:** 2014-11-25

**Authors:** Nazila Tehrani, John Del Rosario, Moises Dominguez, Robert Kalb, Itzhak Mano

**Affiliations:** 1 Physiology Pharmicology & Neuroscience, Sophie Davis School of Biomedical Education, City College of New York (CCNY), The City University of New York (CUNY), New York, New York, United States of America; 2 Graduate Program in Neurobiology, The Graduate Center, The City University of New York (CUNY), New York, New York, United States of America; 3 MA Program in Biology, City College of New York (CCNY), The City University of New York (CUNY), New York, New York, United States of America; 4 Undergraduate Program in Biology, City College of New York (CCNY), The City University of New York (CUNY), New York, New York, United States of America; 5 Neuroscience Graduate Group, Children's Hospital of Philadelphia, University of Pennsylvania, Philadelphia, Pennsylvania, United States of America; Sackler Medical School, Tel Aviv University, Israel

## Abstract

During ischemic stroke, malfunction of excitatory amino acid transporters and reduced synaptic clearance causes accumulation of Glutamate (Glu) and excessive stimulation of postsynaptic neurons, which can lead to their degeneration by excitotoxicity. The balance between cell death-promoting (neurotoxic) and survival-promoting (neuroprotective) signaling cascades determines the fate of neurons exposed to the excitotoxic insult. The evolutionary conserved Insulin/IGF Signaling (IIS) cascade can participate in this balance, as it controls cell stress resistance in nematodes and mammals. Blocking the IIS cascade allows the transcription factor FoxO3/DAF-16 to accumulate in the nucleus and activate a transcriptional program that protects cells from a range of insults. We study the effect of IIS cascade on neurodegeneration in a *C. elegans* model of excitotoxicity, where a mutation in a central Glu transporter (*glt-3*) in a sensitizing background causes Glu-Receptor –dependent neuronal necrosis. We expand our studies on the role of the IIS cascade in determining susceptibility to excitotoxic necrosis by either blocking IIS at the level of PI3K/AGE-1 or stimulating it by removing the inhibitory effect of ZFP-1 on the expression of PDK-1. We further show that the components of the Cytohesin/GRP-1, Arf, and PIP5K/PPK-1 complex, known to regulate PIP2 production and the IIS cascade, modulate nematode excitotoxicity: mutations that are expected to reduce the complex's ability to produce PIP2 and inhibit the IIS cascade protect from excitotoxicity, while overstimulation of PIP2 production enhances neurodegeneration. Our observations therefore affirm the importance of the IIS cascade in determining the susceptibility to necrotic neurodegeneration in nematode excitotoxicity, and demonstrate the ability of Cytohesin/GRP-1, Arf, and PIP5K/PPK-1 complex to modulate neuroprotection.

## Introduction

Stroke/brain ischemia is the fourth leading cause of death in the US [1]. Current therapeutic interventions have very limited success, and pharmacological trials based on previous understanding of the neurodegenerative process ended with disappointment [2]–[5]. In brain ischemia, waves of destruction propagate from the acute center of injury to cause cell death by necrosis and apoptosis, while in the penumbra (the area surrounding the ischemic core), neurons that are initially “stunned” might later die or recover [6]–[9]. The molecular mechanisms that lead to these different fates are not fully understood, but the strongest and largest body of evidence suggests that synaptic accumulation of Glutamate (Glu) and excessive postsynaptic stimulation is a central mediator of toxicity [10]. During ischemia, the clearance of Glu by secondary-active Glu transporters (GluTs) declines [11]–[14], causing synaptic Glu accumulation, overstimulation of ionotropic Glu Receptors (GluRs), and a large influx of Ca^2+^ that might lead to neurodegeneration in a process termed excitotoxicity [4], [15]–[18]. Surprisingly, accumulating evidence indicates that GluR activation contributes to both cell death and neuroprotection [2], [4], but our understanding of both Glu-induced and Glu-independent mechanisms of neuroprotection remains incomplete. We are therefore interested in identifying neuroprotective mechanisms that might regulate the susceptibility of neurons to excitotoxicity.

The evolutionary conserved Insulin/IGF Signaling (IIS) cascade was identified in *C. elegans* as controlling both animal longevity and cell stress resistance [19]–[21]. This cascade includes the nematode Insulin/IGF-1 receptor DAF-2 [22], the PI3-kinase AGE-1 [23], the PIP3- dependent kinase PDK-1 [24], and the protein kinase AKT-1 [25], which controls the phosphorylation of the FoxO3-like transcription factor DAF-16 [26]. Active IIS cascade sequesters DAF-16 in the cytoplasm, while reduced IIS activity allows unphosphorylated DAF-16 to equilibrate to the nucleus, where it controls gene expression [27]–[29]. Mutations that block this pathway confer cell resistance to insults like oxidative stress [30], hypoxia [31], and human-disease-related proteotoxins [32]–[37]. Parallel studies in mammals show that although in some cases FoxO induces apoptosis [38], the IIS pathway confers resistance to non-apoptotic insults [37], [39]. We are therefore interested in the potential of the IIS cascade to mediate cell stress resistance in the excitotoxic scenario, and regulate susceptibility to excitotoxic neurodegeneration.

Cell stress resistance control by IIS is only one of the many signaling pathways conserved from nematodes to humans. Conservation of function extends also to the use of Glu and the molecular building blocks that mediate its function as an excitatory neurotransmitter in the nervous system [40]. We have recently established a model of neurodegeneration in the nematode using a knockout (KO) of the critical GluT gene *glt-3* [41] in the sensitizing background *nuIs5* [42] (expressing hyperactive Gαs and GFP in command interneurons under the *glr-1* promoter). This combination causes extensive neuronal necrosis that is dependent on Ca^2+^-permeable GluRs, defining it as nematode excitotoxicity [43]. Neuronal necrotic corpses appear gradually during development (in correlation with the maturation of Glu signaling in the worm), and peak at the L3 larval stage before they are removed by engulfment. We further used our model of excitotoxicity in *C. elegans* to identify the IIS cascade as a factor that can modulate the extent of neurodegeneration in both nematodes and mammalian neuronal cultures [44]. We observed that FoxO3/DAF-16 provides neuroprotection from excitotoxicity in *glt-3;nuIs5* worms: both a mutation in PI3K/AGE-1 that blocks IIS from expelling FoxO3/DAF-16 from the nucleus, and a drug that translocates FoxO3/DAF-16 into the nucleus reduced the extent of neuronal necrosis in nematode excitotoxicity.

We now look for upstream regulators of IIS in the modulation of excitotoxicity. We are especially intrigued by the function of a complex of proteins that include the Guanine Exchange Factor (GEF) Cytohesin/GRP-1, the small G-protein Arf, and the PIP2-synthesizing enzyme PIP5K/PPK-1. A number of studies in mammals and flies link the Cytohesin/Arf/PIP5K complex to insulin signaling-dependent liver metabolism, membrane transport, and cell growth, demonstrating its functions in providing PIP2 as a substrate for PI3K/AGE-1 and therefore as a stimulator of the IIS cascade [45]–[48]. Indeed, blocking Cytohesin causes a reduction in Akt activation and accumulation of FoxO in the nucleus of both mammalian liver cells and fly S2 cells [45], [46]. We find the Cytohesin/Arf/PIP5K complex to be particularly relevant to our study of excitotoxicity because its components have also been associated with the Post Synaptic Density (PSD) that orchestrates intracellular signaling complexes associated with GluRs. These include a Cytohesin-binding scaffolding protein [49]–[51] that also binds the PSD-organizing protein PSD-95 [52] and metabotropic GluRs [53], [54], and Arf1's association with the GluR-binding protein PICK1 [55]. A few studies address Cytohesin/Arf/PIP5K complex function in *C. elegans*, showing that Cytohesin/GRP-1 and Arf can control asymmetric cell division [56]–[58], and that PIP5K/PPK-1 functions in neurons to produce PIP2 and maintain neuronal development and integrity [59]. In the present study we use both IIS inhibition and stimulation to affirm that suppressing the IIS cascade in *glt-3;nuIs5* animals is neuroprotective in nematode excitotoxicity, and we establish that the IIS-regulating Cytohesin/Arf/PIP5K complex modulates this neuroprotective effect.

## Materials and Methods

### Strains

*C. elegans* strains were generate and maintained using standard methods. Strains used in this study include: **Nematode Excitotoxicity** [43]: ZB1102: *Δglt-3 (bz34) IV; nuIs5 [P_glr-1_::gfp-1;P_glr-1_::Gαs(Q227L) V; lin 15(+)];* ***zfp-1 KO*** [60], [61]: RB774: *Δzfp-1 (ok554) III;* ***grp-1 KO*** [57], [62]: *otIs114 Is [P_lim-6_::gfp; rol-6(d)] I; otIs220 Is [P_gcy-5_::mCherry; rol-6(d)] IV; grp-1 (tm1956) III* (we preserved only the *grp-1* mutation during the cross with the excitotoxicity strain); ***arf-1.2 KO*** [57], [63]: VC567: *Δarf-1.2 (ok796) III;* ***ppk-1 Over Expression*** [59]: *EG3361 (lin-15(n765ts) X oxIs12 [P_unc-47_::GFP, lin-15+] X, gqIs25 [P_rab-3_::ppk-1, lin-15(+)] I.* (*oxIs12 [P_unc-47_::GFP, lin-15+] X* was eliminated during the cross with our excitotoxicity strain, while *gqIs25* was preserved). ***ced-4*** [64]: MT2551 *ced-4(n1162) dpy-17(e164)III*. Some strains were obtained from The Caenorhabditis Genetics Center (CGC, the University of Minnesota) and the Japanese National Bioresource Project (NBRP, Tokyo Women's Medical University School of Medicine). For genotyping, deletions were followed by PCR, and *nuIs5* was followed by the presence of *P_glr-1_::GFP*. *ced-4* was followed initially by the linked *dpy* phenotype and then confirmed by sequencing the *n1162* allele. To identify animals carrying the *P_rab-3_::PPK-1* over expressing construct we performed a PCR amplification of a fragment that detects this fusion construct, using a 5′ primer from the *rab-3* promoter region and a 3′ primer from the *ppk-1* genomic sequence. These primers give a ∼400 bp product observed only in *gqIs25[P_rab-3_::PPK-1]* animals.

### Neurodegeneration quantification

**Figure 1:**
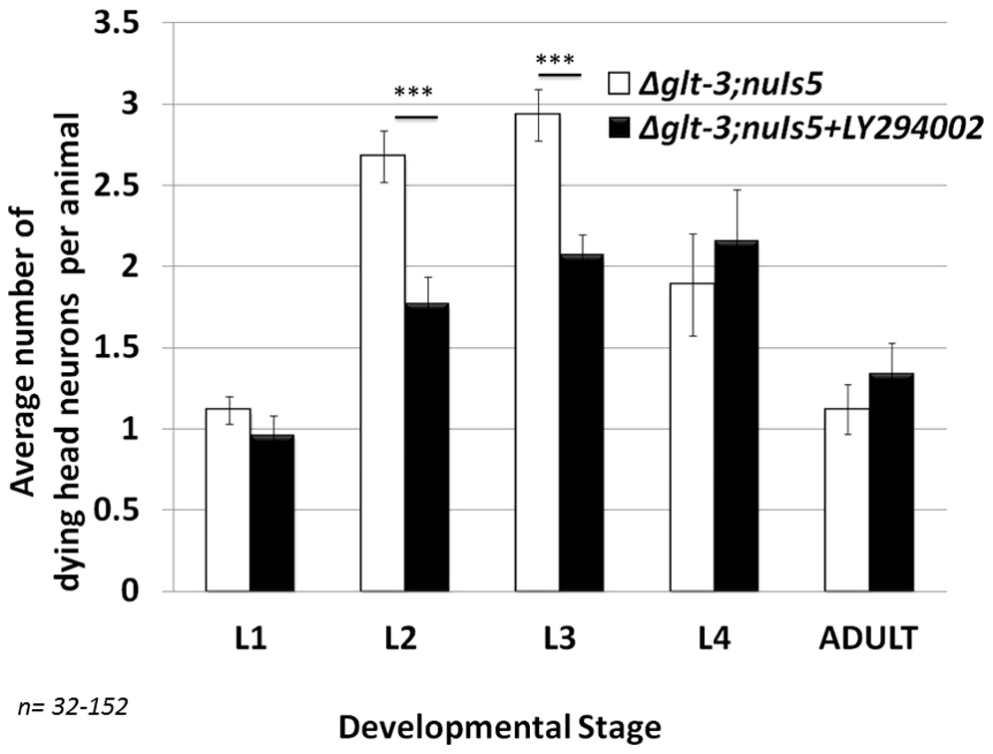
LY294002, an inhibitor of PI3K/AGE-1, confers neuroprotection in nematode excitotoxicity. Sham (ethanol only) treated or LY294002 (ethanol + drug) treated animals were scored for neuronal necrosis throughout development. Neurodegeneration scoring is described in Materials & Methods. In all histograms, error bar represent SE. ***: p<0.01.

**Figure 2:**
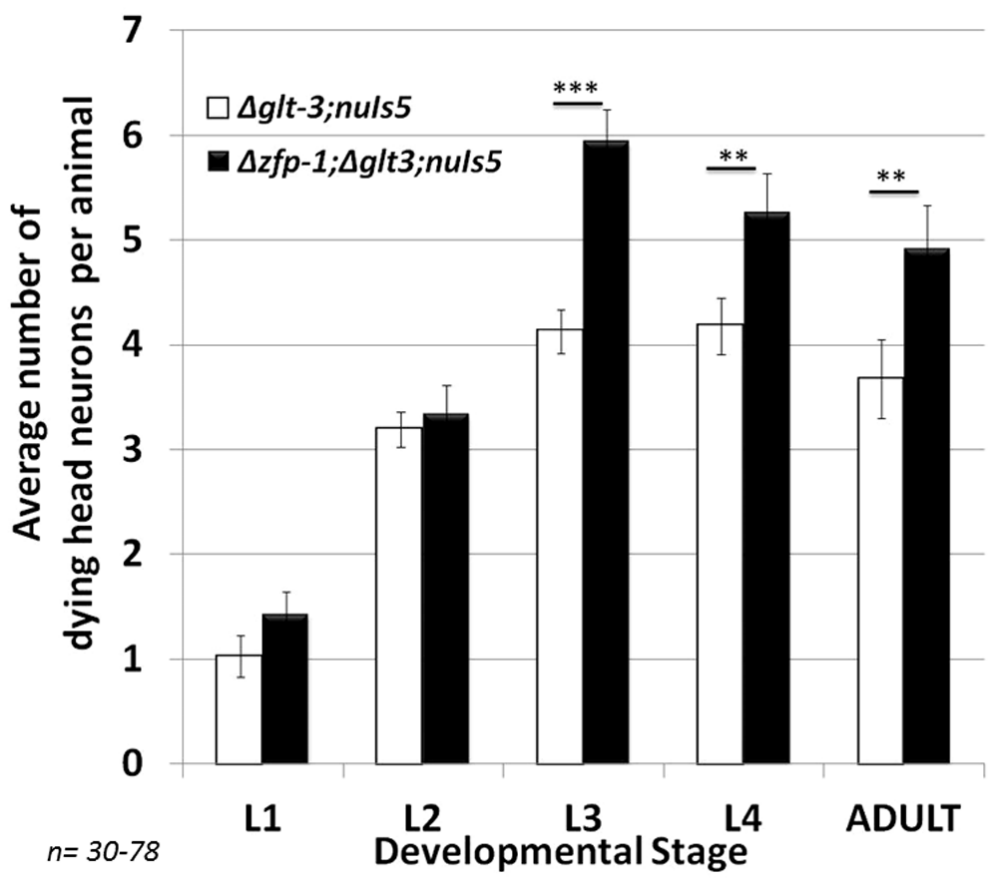
KO of *zfp-1*, an inhibitor of PDK-1 transcription, exacerbates nematode excitotoxicity. **: p<0.05; ***: p<0.01.

**Figure 3:**
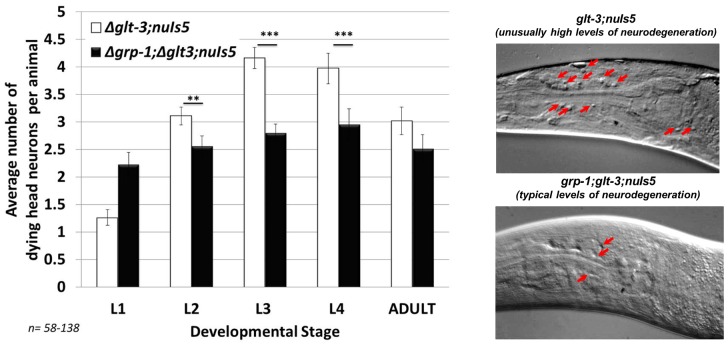
KO of *grp-1*, a GEF that stimulates Arf and PIP5K/PPK-1 to increase production of PIP2 substrate for the IIS cascade, provides neuroprotection. Left: a histogram showing a decrease in neurodegeneration upon KO of *grp-1* (a second independent cross gave very similar distribution, not shown) **: p<0.05; ***: p<0.01. Right: Nomarski/DIC images of neurodegeneration in head neurons. Anterior left, dorsal top, the nerve ring area is shown (located between the two bulbs of the pharynx), red arrows indicate degenerating neurons. A typical level of neurodegeneration in our excitotoxicity strain was depicted previously [43]. We note that the extent of neurodegeneration varies among individual animals of the same genotype. Here, the upper image depicts an individual L3 animal from our excitotoxicity strain with an unusually high level of neurodegeneration. The extensive neurodegeneration seen in such untypical animals is evened out in the large number of animals used for each bar in our histograms (usually >90 animals in the most informative stages), bringing average neurodegeneration levels in our excitotoxicity strain (*glt-3;nuIs5*) to ∼4.5 dying head neurons/L3 animal. The bottom image depicts a typical *grp-1;glt-3;nuIs5* L3 animal, a strain that typically shows 2–3 dying head neurons/L3 animal.

**Figure 4:**
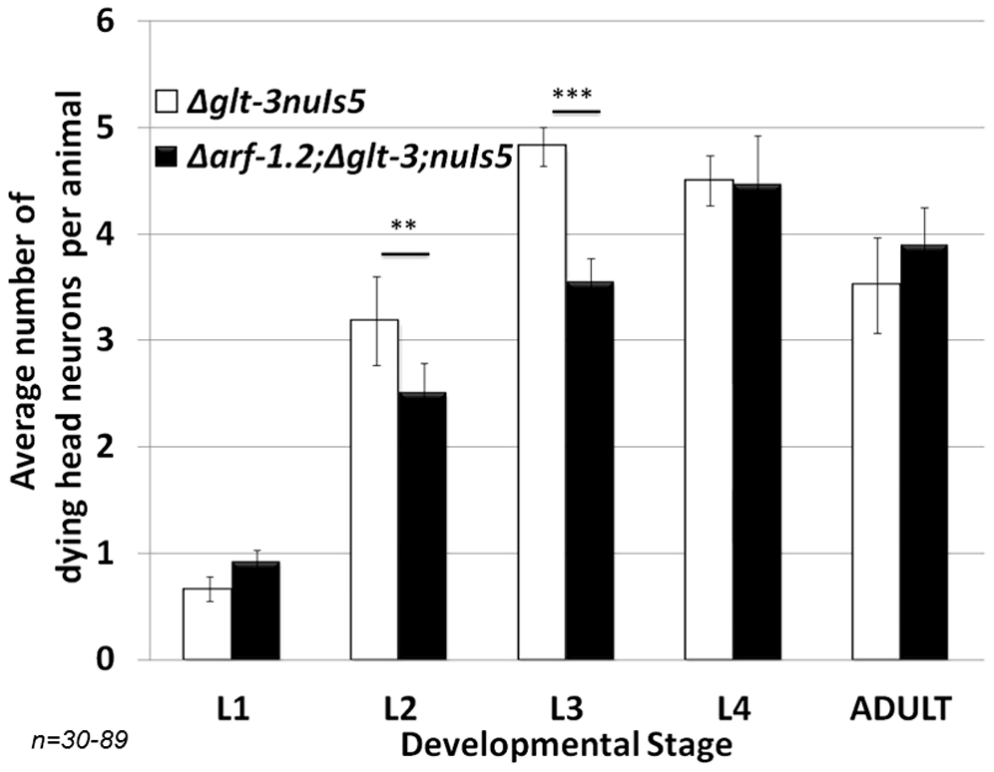
KO of *arf-1.2* provides neuroprotection. **: p<0.05; ***: p<0.01.

**Figure 5:**
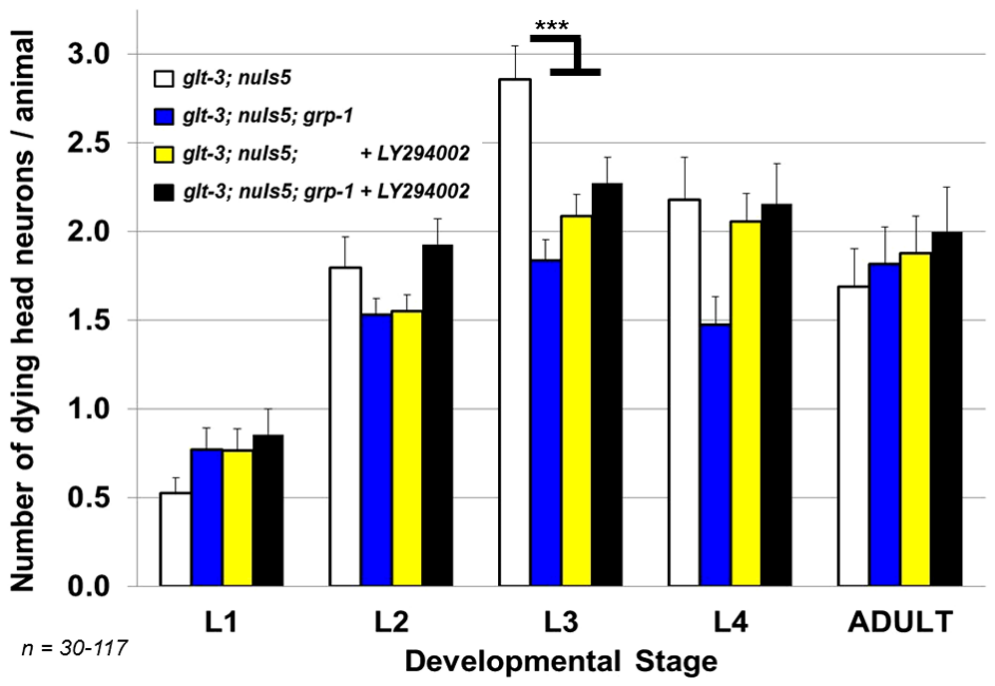
Epistasis analysis suggests that *grp-1* works in the same pathway as *age-1*. *grp-1* was inactivated using a KO strain. *age-1* was inhibited using the drug LY294002. If these two factors worked in separate pathways, their ability to suppress neurodegeneration would be (at least partially) additive, a concept not supported by our observations. The levels of neurodegeneration seen in our original excitotoxicity strain (under ethanol conditions needed to be used in this experiment) is equally different from the reduced neurodegeneration seen with inhibition of *grp-1*, *age-1*, or both (***: p<0.01).

**Figure 6:**
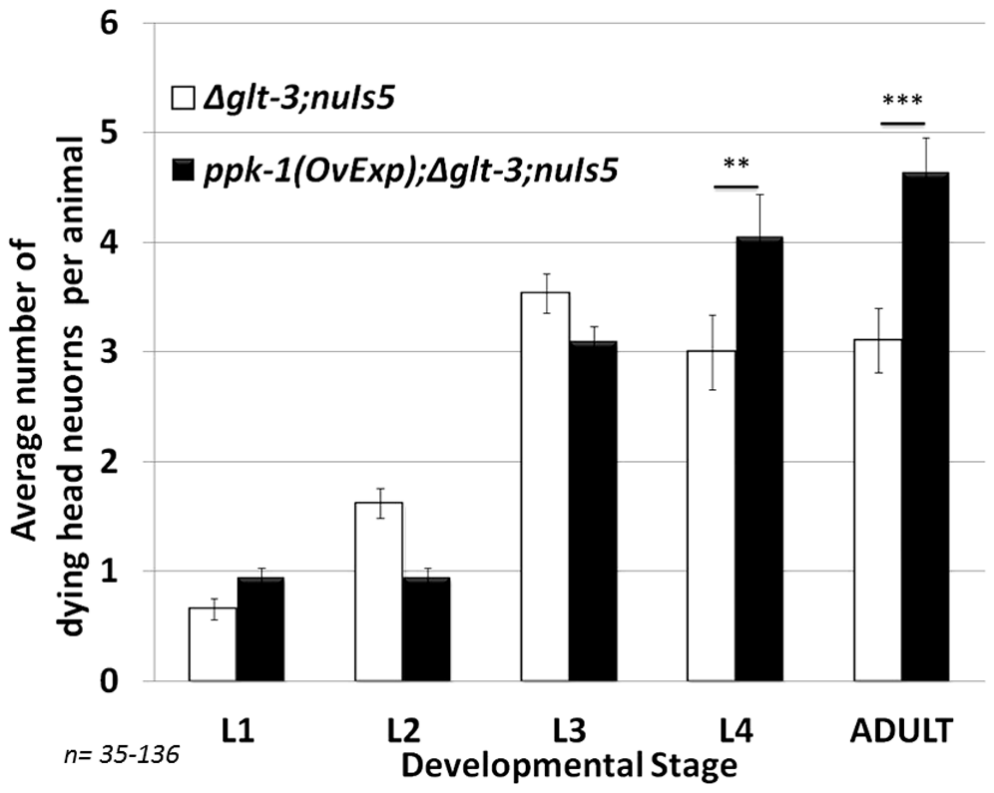
Over Expression of PPK-1, known to lead to over-production of PIP2, exacerbates necrotic neurodegeneration. **: p<0.05; ***: p<0.01.

**Figure 7:**
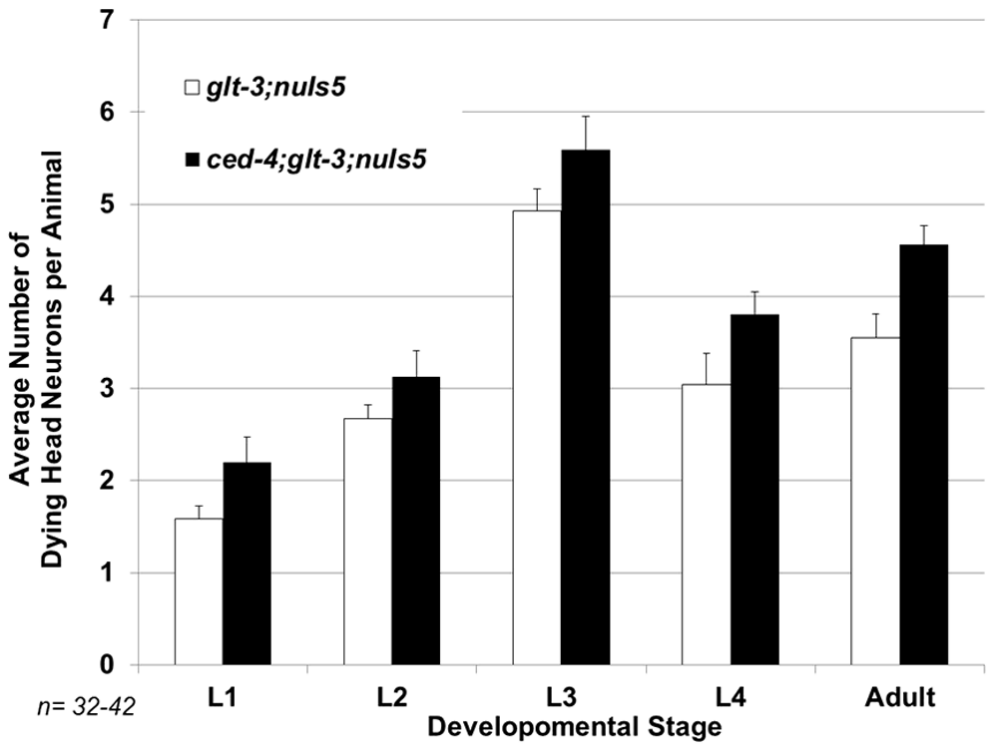
Blocking canonical apoptosis using a *ced-4* mutation does not suppress cell death in nematode excitotoxicity.

Levels of excitotoxic neurodegeneration were quantified as described by Mano & Driscoll [43] and in line with standard methods used in studies of other forms of necrotic neurodegeneration in *C. elegans* [65], [66]. All neurodegeneration studies were performed on strains that contain the excitotoxicity-producing combination of *glt-3;nuIs5* (without or with additional mutations). Animals were mounted with an agar chunk on a cover slip and observed using an inverted DIC microscope (without anesthesia). The animals on the chunk were screened, individual animals were classified for their developmental stage, and the number of degenerating neurons for each animal was recorded. Necrotic neurodegeneration is seen as swollen neurons that look like vacuolated structures (occasionally verified to correspond to *nuIs5/P_glr-1_::gfp*-expressing cells). Similarly to the stochastic nature of neuronal necrosis seen with other triggers of necrotic neurodegeneration in *C. elegans* (and unlike the more constant developmental apoptotic cell death), the number of degenerating neurons in the control group is not stereotypically repeated in exact values (an effect that is further compounded by the fact that not all of the ∼30 *glr-1* -expressing “at-risk” neurons ultimately die by adulthood). Instead, cell death shows a very typical dynamics, as it peaks at L3 with the maturation of Glu signaling in the worm, and then goes down as cell corpses are engulfed and removed. The level of neurodegeneration in our excitotoxicity model can vary in response to growth conditions, and keeping the strain running by repeated re-chunking over very long periods can suppress its levels. Therefore, special care was given to the use of recently isolated or outcrossed strains, the use of freshly grown (non-stressed) animals in multiple sessions, and in each session, comparison of test strains to control animals exposed to identical growth conditions (thus controlling for variations between experiments, similarly to standard practice in nematode lifespan experiments). Each bar in figures 1–7 corresponds to at least 30 animals, with over 90 animals usually scored at L3. As per standards in the nematode necrotic neurodegeneration field, error bars represent SE. Statistical significance of difference between strains is measured using z score, and is indicated on the graph whenever the difference is significant. Whenever possible, the basic excitotoxicity strain (*glt-3;nuIs5*) used as reference in each experiment was re-isolated from the new cross, to enhance the similarity with the new strain being tested. Critical new strains were obtained in two independent crosses and neurodegeneration was scored to verify the effect in independent strains.

### LY294002 treatment

LY294002 (LC Laboratories) drug was dissolved in 100% ethanol to produce a stock solution of 25 mM. 20 microliter of ethanol without (control) or with LY294002 were added to 12 well plates with MYOB agar+OP50 bacteria [67] to produce final concentration of 0.2 mM. After ethanol was absorbed, the worms were added to these culture plates. After 3 days, the level of neurodegeneration in head neurons was determined. Worms were kept on fresh drug/control by chunking them to fresh plates with the appropriate condition (ethanol only or ethanol+LY294002) and were used for additional sessions of neurodegeneration scoring. Since ethanol has an inhibitory effect of the basic level of excitability in *C. elegans* [68], extra caution was taken to verify the validity of the LY294002 effect under these conditions. These sets of experiments were run several times, with large number of animals counted in each one. Figure 1 shows one of these experiments, with the other ones giving very similar results and an identical trend.

## Results

### A widely used method of chemical inhibition of the IIS pathway confers neuroprotection from excitotoxic neurodegeneration in *C. elegans*

A number of studies in mammalian cells suggest that blocking the IIS cascade and AKT activation enhances neuronal apoptosis in excitotoxicity [4], [69]–[73], while our previous studies in both nematodes and mouse neuronal cultures suggest that blocking the IIS cascade reduces excitotoxic necrosis [44]. Most of the mammalian studies attributing a neuroprotective/anti-apoptotic effect to Akt stimulation used the PI3K inhibitor LY294002 to inhibit IIS and Akt activation, a drug that also shows IIS-blocking effects in *C. elegans* [74]. To address this possible controversy and further verify that blocking the IIS pathway in nematodes results in reduced excitotoxic necrosis we monitored the effect of the LY294002 on nematode excitotoxicity in *glt-3(bz34);nuIs5* animals (Figure 1). Exposing *glt-3;nuIs5* animals to the ethanol used to dissolve this drug (without applying the drug itself) causes a moderate reduction in the number of necrotic corpses in head neurons compared to non-treated animals (in line with the reported effects of ethanol exposure on neuronal excitability in nematodes [68]). However, the overall pattern of necrosis during development in these sham-treated animals remains similar to that of non-treated *glt-3;nuIs5* animals. Importantly, the application of LY294002 caused a significant reduction in excitotoxic necrosis compared to sham treated animals, reducing neurodegeneration from an average of 3 degenerating head neurons per animal without the drug to 2 head neurons per animal in the presence of LY294002. These observations reaffirm that a variety of treatments that reduce the activity of the IIS cascade activity are neuroprotective in nematode excitotoxicity.

### Genetic stimulation of the IIS cascade by zfp-1 mutation increases susceptibility to nematode excitotoxicity

A particularly strong approach in genetic analysis of signaling cascades is to demonstrate that over-activation of the cascade leads to an opposite phenotype than its inhibition. To solidify our understanding of the role of the IIS cascade in nematode excitotoxicity we therefore studied the effect of its over-activity. The transcription regulator and AF10 homolog ZFP-1 [61], [75], [76] provides a particularly interesting opportunity, since it exerts strong regulation over the IIS cascade. Transcription of the *zfp-1* gene is moderately stimulated by FoxO3/DAF-16 [77], [78]. More importantly for our analysis, ZFP-1 itself is a strong inhibitor of the IIS cascade: ZFP-1 acts (together with DOT-1) to reduce histone modification at specific genes and prevent their transcription during stress response [75]. A prime target of ZFP-1-mediated transcriptional suppression is the gene encoding the IIS protein PDK-1 (which normally functions to activate AKT in response to PI3K/AGE-1 stimulation). Therefore, under stress conditions ZFP-1 normally inhibits PDK-1 expression, leading to increased DAF-16 –mediated stress resistance. In *zfp-1* mutant animals PDK-1 expression goes uninhibited, the IIS cascade is overactive, and DAF-16-mediated stress resistance is reduced [78]. We therefore tested the effect of *zfp-1* mutation on the susceptibility to excitotoxic stress. We find that the *zfp-1(ok554)* mutation indeed causes increased susceptibility to excitotoxicity, increasing the average number of necrotic neurons in the L3 stage from 4 to 6 (Figure 2). We therefore affirm that active IIS increases susceptibility to neurodegeneration while treatments that activate FoxO3/DAF-16 protects from neuronal necrosis in nematode excitotoxicity.

### Mutations in Cytohesin/GRP-1 and ARF-1.2, expected to reduce IIS signaling, confer neuroprotection from excitotoxicity

We next investigated the role of the Cytohesin/GRP-1, Arf, and PIP5K/PPK-1 complex, known to regulate PIP2 production and the IIS cascade, in nematode excitotoxicity. We used genetic analysis, combining the excitotoxicity genetic background (*glt-3;nuIs5*) with mutations that affect this complex. This approach is usually more productive in *C. elegans* than pharmacological intervention (which many time is ineffective in the worm) or RNAi (which many times is ineffective in nematode neurons), though it has its drawbacks. For example, there are a few Arf homologs in the worm, but only some can be studied by genetic elimination, since their KO strain is lethal (as is *ppk-1 KO*). However, we managed to study the KO of two key components [57], [58], [63]: the GEF Cytohesin/GRP-1 and the small G-protein ARF-1.2. Since both Cytohesin/GRP-1 and Arf stimulate the activity of the PIP-2 synthesizing enzyme PIP5K/PPK-1, their KO is expected to reduce PIP5K/PPK-1 activity, reduce the supply of PIP2 to the IIS cascade and inhibit its activity, leading to an increase in cell stress resistance. Indeed, in both cases, KO of either *grp-1* (using the *tm1956* allele) (Figure 3) or *arf-1.2* (using the *ok796* allele) (Figure 4) suppressed neurodegeneration in nematode excitotoxicity.

### Modulation of excitotoxic neurodegeneration by GRP-1 is exerted through the IIS pathway

To verify that the ability of GRP-1 elimination to reduce excitotoxic neurodegeneration is mediated through the IIS cascade we blocked the IIS cascade in *glt-3;nuIs5* animals using LY294002, and compared animals that have WT *grp-1* to animals carrying a *grp-1 KO*. Neurodegeneration levels in *grp-1;glt-3;nuIs5* animals exposed to LY294002 was very similar to that of *glt-3;nuIs5* animals exposed to LY294002 (Figure 5). These observations suggest that GRP-1 mediates its action on excitotoxic neurodegeneration through the IIS cascade, and inhibiting the cascade with both a *grp-1* mutation and LY294002 has no additional neuroprotective effect.

### Over expression of the PIP5K/PPK-1, known to cause excessive production of PIP2, exacerbates excitotoxic neurodegeneration

To circumvent the challenge of the lethality of *ppk-1 KO* mutant and to induce a hyperactivation of the Cytohesin/GRP-1 – PIP5K/PPK-1 complex (and the IIS cascade) we used a strain that exhibits over-expression and excessive activity of PPK-1. Weinkove *et al.* found that over-expressing PPK-1 from the powerful pan-neuronal *rab-3* promoter causes excessive production of PIP2, and that mature neurons are especially susceptible to PPK-1 overexpression [59]. If PPK-1 supplies the PIP2 substrate for the IIS cascade, then overexpression of PPK-1 should overstimulate the IIS cascade and cause excessive neurodegeneration. Indeed, when we introduced the *P*_rab-3_ *::PPK-1* construct to *glt-3;nuIs5* animals we saw an increase in the level of necrotic neurodegeneration (Figure 6). The necrotic effect of PPK-1 hyperactivation is seen a bit later in development than our usual peak at L3, appearing instead when the *P_rab-3_:PPK-1* construct produces its full effect [59]. Together with the data on GRP-1 and ARF-1.2, these observations suggest that the IIS-stimulating complex of Cytohesin/GRP-1, Arf, and PIP5K/PPK-1 serves to increase susceptibility to excitotoxicity in the nematode.

### Nematode excitotoxicity is not affected by a mutation in ced-4

To increase the validity of our conclusion that the Cytohesin/GRP-1, Arf, and PIP5K/PPK-1 complex regulates the IIS cascade to determine the level of susceptibility to excitotoxicity, we also tested other possible explanations for the neuroprotective effect of *grp-1* mutation. One alternative explanation is that the IIS cascade directly regulates the level of expression of GluRs. Our initial observations using a synaptically localized GLR-1 or behavioral assays do not provide support for a strikingly large change in GLR-1 expression level, though these studied are not yet conclusive (data not shown).

Another alternative explanation for the effect of *grp-1* on the level of excitotoxic neurodegeneration is based on the involvement of *grp-1* in apoptosis, as seen in some post-embryonic lineages in the nematode [58]. If apoptosis mediates or participates in some of the cell death we see in excitotoxic neurodegeneration in the nematode, a mutation in an apoptosis regulator such as *grp-1* could reduce the extent of cell death. To test the possible involvement of apoptosis as a mediator of neurodegeneration in our excitotoxicity model we blocked apoptosis using the *ced-4(n1162)* mutation [64]. However, similarly to the lack of involvement of apoptosis in *mec-4(d)* –induced necrosis [79], the mutation in *ced-4* did not affect the level of excitotoxic neurodegeneration (Figure 7). We therefore conclude that canonical apoptosis does not play a significant role in the condition that we study, and therefore cannot explain the ability of Cytohesin/GRP-1 mutation to inhibit neurodegeneration in nematode excitotoxicity.

## Discussion

### Activation of the IIS cascade increases susceptibility to nematode excitotoxicity

The role of the IIS cascade in excitotoxic neurodegeneration seems to be controversial. A large number of mammalian studies conclude that AKT activation is neuroprotective, while FoxO3 activation increases apoptotic neurodegeneration in a variety of conditions including excitotoxicity [4], [69]–[73]. In contrast, other studies in nematodes and mammals point to a strong neuroprotective function for IIS cascade inhibition and DAF-16/FoxO3 activation. Our data on nematode excitotoxicity (and previously also in mammalian primary cultures [44]) support the neuroprotective view for DAF-16/FoxO3 activation. We now reaffirm our previous observation by using LY294002, the same drug that was used in the mammalian studies, showing that it causes neuroprotection (Figure 1). We also hyperactivated the IIS cascade using the *zfp-1* mutation and observed excessive necrosis (Figure 2). We are therefore convinced that an active IIS cascade increases susceptibility to excitotoxic necrosis in *C. elegans*, and its inhibition leads to neuroprotection. We do not have a full explanation to the difference in opinions in the field, other than difference in experimental setup and the characterization of cell death. Indeed, one clear difference between our study and previous ones is that we focus very specifically on necrotic cell death in excitotoxicity, while many other studies might involve several death mechanisms or focus on apoptotic cell death. The condition that we study does not seem to involved apoptosis (Figure 7). The ability of FoxO activation to lead to diverse consequences, depending in the exact combination of cellular factors, is well documented [38], [80]. We therefore suggest the simplified scenario of nematode excitotoxicity, where apoptosis is not involved, allows us to clearly dissect a neuroprotective effect for FoxO/DAF-16, an effect that participates also in (at least some of-) the more complex scenarios that take place in mammalian excitotoxicity (as seen in our previous study [44]). In the future, this might help us illuminate conserved neuroprotection-specific processes in excitotoxicity downstream of FoxO/DAF-16.

### The IIS-stimulating complex of GRP-1 & PPK-1 serves to regulate excitotoxicity

**Figure 8:**
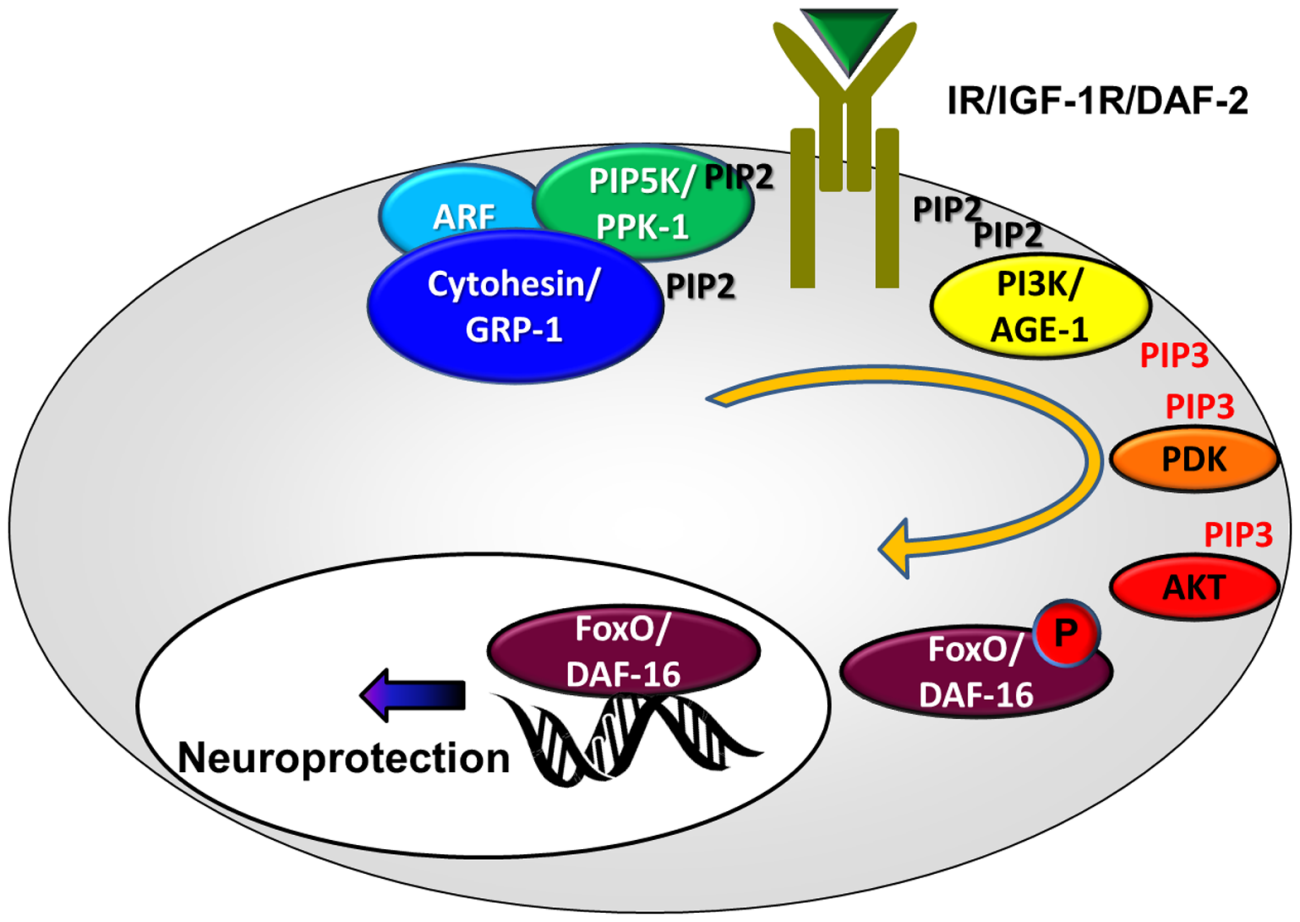
A model for regulation of IIS-mediated neuroprotection in nematode excitotoxicity. When the Cytohesin/GRP-1, Arf, and PIP5K/PPK-1 complex is active, it stimulates the IIS cascade, resulting in phosphorylation and cytoplasmic sequestration of FoxO/DAF-16. If the IIS cascade is less active, un-phosphorylated FoxO/DAF-16 accumulates in the nucleus and activates a transcriptional program that results in neuroprotection from the excitotoxic insult.

Our data puts the spotlight on the IIS-regulating Cytohesin/GRP-1, Arf, and PIP5K/PPK-1 complex and its role in regulating susceptibility to excitotoxicity in *C. elegans*. Using epistasis we demonstrate that *grp-1* works in the same pathway as *age-1* to regulate neurodegeneration levels. We further show that this effect is unlikely to involve *grp-1*'s regulation of apoptosis (seen in some neuronal lineages), as apoptosis seems not to be involved in nematode excitotoxicity. It is possible that other IIS cascade-regulated processes might also be influenced by this complex. However, as the focus of our research is excitotoxicity, our data does not address those other functions of the IIS cascade. Together with our previous data on the nuclear translocation of DAF-16 as a means to induce neuroprotection, our studies are therefore in line with a model where the Cytohesin/GRP-1, Arf, and PIP5K/PPK-1 complex controls the transcriptional output of the IIS cascade to regulate susceptibility to excitotoxicity (Figure 8).

### The GRP-1 & PPK-1 might serve as a link that allows GluR to control neuroprotection and susceptibility to excitotoxicity

Our initial interest in the Cytohesin/GRP-1, Arf, and PIP5K/PPK-1 complex was based on the studies that indicate its physical association with the PSD and with GluRs. Currently the subcellular localization of this complex is unknown (other than the observation by Weinkove *et al.* [59] that PPK-1 is expressed throughout the cell membrane of all neurons, and could therefore overlap with expression of GluRs in post-synaptic areas of the neurites). It also remains to be seen if GluRs provide any input to IIS signaling via the Cytohesin/GRP-1, Arf, and PIP5K/PPK-1 complex. It should be noted that ample evidence exists in mammals for a functional interaction between GluRs and insulin signaling [81]–[84]. Some of these studies describe a rapid effect of insulin receptors on GluR distribution [85]–[88]. Interestingly, a seminal study shows that a phosphatase that degrades PIP3 is associated with the PSD and serves to suppress excitotoxic neurodegeneration [89], a scenario that is in line with our model. For the time being we do not know if some of the neuroprotective or neurotoxic effects of Glu are mediated by GluR-IIS cross talk that regulates neuroprotection by FoxO/DAF-16. Therefore it is not clear if the level of IIS signaling is a “pre-existing condition” that determine susceptibility to neurodegeneration, or if it can be actively modified by Glu signaling, providing an important venue for Glu to control both neurodegeneration and cell survival.
